# An integrated network representation of multiple cancer-specific data for graph-based machine learning

**DOI:** 10.1038/s41540-022-00226-9

**Published:** 2022-04-29

**Authors:** Limeng Pu, Manali Singha, Hsiao-Chun Wu, Costas Busch, J. Ramanujam, Michal Brylinski

**Affiliations:** 1 Center for Computation and Technology, Louisiana State University, Baton Rouge, LA 70803 USA; 2 Department of Biological Sciences, Louisiana State University, Baton Rouge, LA 70803 USA; 3 Division of Electrical and Computer Engineering, Louisiana State University, Baton Rouge, LA 70803 USA; 4 Division of Computer Science and Engineering, Louisiana State University, Baton Rouge, LA 70803 USA; 5 School of Computer and Cyber Sciences, Augusta University, Augusta, GA 30912 USA

**Keywords:** Molecular medicine, Computational biology and bioinformatics

## Abstract

Genomic profiles of cancer cells provide valuable information on genetic alterations in cancer. Several recent studies employed these data to predict the response of cancer cell lines to drug treatment. Nonetheless, due to the multifactorial phenotypes and intricate mechanisms of cancer, the accurate prediction of the effect of pharmacotherapy on a specific cell line based on the genetic information alone is problematic. Emphasizing on the system-level complexity of cancer, we devised a procedure to integrate multiple heterogeneous data, including biological networks, genomics, inhibitor profiling, and gene-disease associations, into a unified graph structure. In order to construct compact, yet information-rich cancer-specific networks, we developed a novel graph reduction algorithm. Driven by not only the topological information, but also the biological knowledge, the graph reduction increases the feature-only entropy while preserving the valuable graph-feature information. Subsequent comparative benchmarking simulations employing a tissue level cross-validation protocol demonstrate that the accuracy of a graph-based predictor of the drug efficacy is 0.68, which is notably higher than those measured for more traditional, matrix-based techniques on the same data. Overall, the non-Euclidean representation of the cancer-specific data improves the performance of machine learning to predict the response of cancer to pharmacotherapy. The generated data are freely available to the academic community at https://osf.io/dzx7b/.

## Introduction

Carcinogenesis is a systems-level phenomenon with a complex phenotype concomitant with the malfunction of signal transduction in a cell^1–3^. Biological networks, including protein-protein interaction (PPI) networks, are often employed to study the alteration of information flow in cancer cells caused by oncogenic changes in protein activity and expression^4–6^. Many early network-based methods build on the observation that gene products associated with similar diseases exhibit similar topological characteristics of PPI networks. For instance, Vavien utilizes the information flow in networks to extract functional disease-gene relationships in order to identify target genes for pharmacotherapy^7^. Systematic benchmarks of Vavien against the Online Mendelian Inheritance in Man database demonstrated that disease-gene candidates can effectively be prioritized based on their topological similarity to known disease-associated genes. A more recent study comprehensively analyzed 150 anticancer drugs approved by the US Food and Drug Administration divided into two groups according to their mechanism of action, cytotoxic and target-based agents^8^. It was found that although the proportion of target-based drugs increased in recent years, cytotoxic agents are used to treat more cancer types. Further, the investigation of the cancer-drug-target network comprising multiple cancer types, drugs, and targets revealed novel drug-cancer associations, most of which are supported by at least one clinical trial study.

These studies exemplify the utility of biological networks to address challenging tasks in cancer research, such as the efficacy prediction for anticancer drugs. Not surprisingly, there is a significant interest in the development of machine-learning systems applicable to graph-structured data^9^ in order to further improve the information extraction and induction from biological networks. Graph-based machine-learning approaches can broadly be categorized into two major classes, graph kernels and spectral methods. An exemplar of the former technique is the Weisfeiler–Lehman (WL) algorithm^10^, which iteratively assigns a label to each node based on the multi-set hashing of the neighbor labels. Subsequently, graph-level features are computed from either a histogram or another form of summarizing statistics for individual nodes. On the other hand, spectral methods utilize the graph Laplacian, a matrix calculated from the adjacency matrix of a graph^11^. A variety of matrix-related operations, such as eigenvalue decomposition, can be applied to the Laplacian to cluster nodes into different groups and perform graph classification. Alternative techniques to classify nodes and compute various graph statistics include PageRank, a random walk-based algorithm proposed by the founders of Google^12^. This technique determines the importance of a page, represented as a node in the graph, by counting the number and quality of links under the assumption that more important websites receive more links from other websites.

Recently developed graph-based machine-learning approaches for applications in biology and biomedicine include a new prediction method combining multiple kernels into a tripartite, heterogeneous drug-target-disease interaction space in order to integrate multiple sources of biological information^13^. This novel network-based algorithm adds a disease layer to the traditional drug-target interaction bipartite graph. From the constructed heterogeneous network, new drug-target interactions can be inferred with Gaussian kernel functions and the regularized least square method of the Kronecker product. Encouragingly, comprehensive benchmarking simulations demonstrated that the network topology can be used to accurately detect drug-target interactions. Another example is a machine-learning framework to identify robust drug biomarkers with network-based analyses of the pharmacogenomic data derived from three-dimensional organoid culture models^14^. These biomarkers were shown to reliably predict responses of colorectal cancer patients to 5-fluorouracil and bladder cancer patients to cisplatin. Finally, biomarkers were confirmed against the external transcriptomic datasets of drug-sensitive and drug-resistant isogenic cancer cell lines, demonstrating that combining the application of gene modules and network-based techniques can be used to predict anticancer-drug efficacy in patients.

A major challenge for the application of machine-learning systems to biological networks is to ensure that these data contain a sufficiently high signal-to-noise ratio for the learning framework to efficiently perform information extraction and high-level induction. In addition, the size of many biological networks, such as PPI networks comprising thousands of nodes, is prohibitively large for many algorithms and, therefore, should preferably be reduced to facilitate a fast-learning process. To address these challenges, we describe a novel procedure to construct compact yet information-rich, cancer-specific graphs by integrating multiple heterogeneous data on differential gene expression, drug profiling, protein-protein interactions, and disease association scores. The resulting networks are characterized by various graph-based statistics and their information content is evaluated with topological and feature entropy measures. Finally, we conduct a comparative analysis of the performance of matrix- and graph-based predictors of the drug efficacy employing a tissue level cross-validation protocol. Overall, the integrated network representation of heterogeneous biological data improves the performance of machine learning to predict the effect of a drug treatment on the cancer cell growth over more traditional matrix-based approaches.

## Results

### Construction of cancer-specific networks

**Fig. 1:**
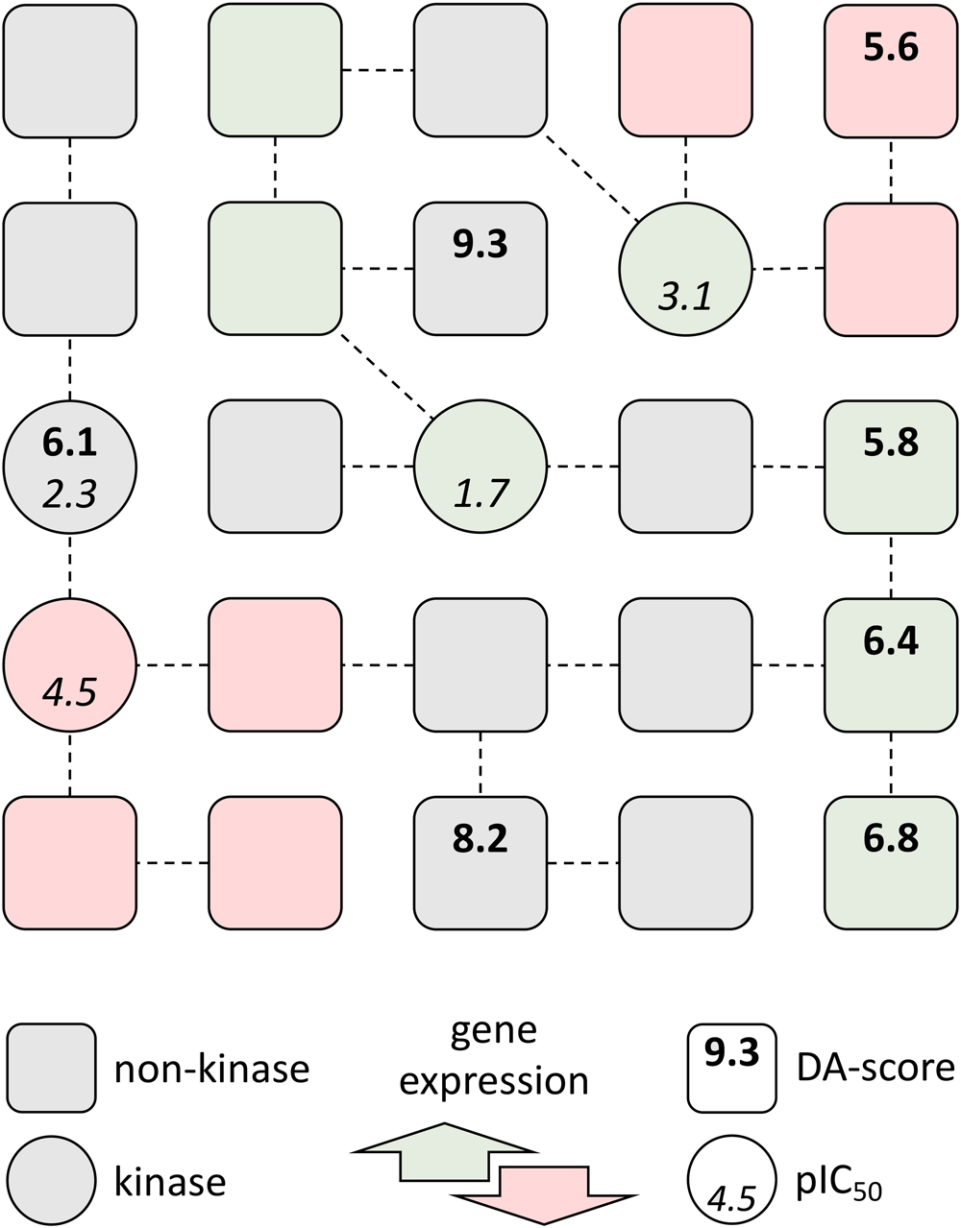
Schematic of the graph representation of multiple heterogeneous data. Circles are kinases, whereas rounded squares represent non-kinase proteins. Nodes are connected through confident interactions forming a network. Each node is colored according to the differential gene expression: green—up-regulated, red —down-regulated, and gray—normally regulated. Both types of nodes can have gene-disease association scores (numbers in bold), whereas kinases can also have pIC_50_ values according to the kinase profiling data (numbers in italics).

Integration of the heterogeneous, cancer-specific data was performed by mapping the differential gene expression, disease-gene association scores, and kinase inhibitor profiling onto the human PPI network. The PPI network contains the majority of proteins and connections of the human phosphorylation network, yet it is larger, more diverse and strongly connected^15^. The integration procedure is schematically shown in Fig. 1. An initial network has two types of nodes representing kinases (circles) and non-kinase proteins (rounded squares). For a given cell line, up- (green) and down-regulated (red) genes are marked according to the differential gene expression for that cell line and some nodes are also assigned disease-gene association scores (numbers in bold). If this cell line is treated with a kinase inhibitor, pIC_50_ values against its targets are then added to the graph (numbers in italics). In the resulting graph, kinase nodes have gene expression values, and some kinase nodes also have pIC_50_ values and disease-gene association scores. Non-kinase nodes have gene expression values, and some non-kinases nodes also have disease-gene association scores. Note that all cell line-drug combinations have the same underlying PPI network, however, different cell lines usually have different gene expression values and disease association scores that depend on the tumor type. Similarly, various drugs usually inhibit different sets of kinases, therefore, node features are generally unique for cell line-drug combinations.

### Analysis of full-size networks

In the context of machine learning, full-size graphs corresponding to the original PPI networks are not necessarily the best representation of the cancer-specific data. First, all instances share the same graph topology and differences are only in node features, i.e., gene expression, disease association, and pIC_50_ values, making it difficult for a machine-learning model to gather the information required for effective learning. Second, full-size graphs are very sparse with a density (see Eq. 1) as low as 0.004 wasting the computer memory. A quick fix to this problem could be to employ a sparse representation, such as the coordination (COO) format^16^, however, this approach is unworkable because available machine-learning libraries do not support sparse data formats. Third, the majority (98%) of nodes in a graph are non-kinase proteins with no inhibition data and most proteins are normally regulated according to the differential gene expression leading to a significant sparsity of important features. As a result, most entries in the feature matrix carry no effective information, which may result in a poor learning performance. An illustrative analogy is to try training a model to classify images having less than 1% different pixels. Given such tiny differences, any model is going to struggle learning the underlying patterns.

### Knowledge-based reduction of cancer-specific networks

In order to address these issues, we devised a knowledge-based graph reduction procedure by edge contraction, which is a fundamental operation in graph theory^17^. Here, the idea is to remove a particular edge and then merge the incident nodes of that edge to form a new node. Edge contraction is widely used in recursive formulas to calculate the number of spanning trees of an arbitrary connected graph^18^, and in recurrence formulas to calculate the chromatic polynomial of a simple graph^19^. Nonetheless, a simple edge contraction based solely on the connectivity is not going to produce the desired outcome in our case because we also need to account for the features of nodes. Therefore, we developed a knowledge-based edge contraction algorithm employing both connectivity and biological feature information to satisfy the following conditions: both incident nodes need to be non-kinase proteins, share the same differential gene expression, and belong to the same biological process cluster. The last condition is very important to ensure that the reduction merges only those nodes belonging to the same pathway, thus supporting the biological knowledge. Biological processes in cancer-specific networks are determined by clustering nodes according to the similarity of their Gene Ontology (GO) terms.

**Fig. 2:**
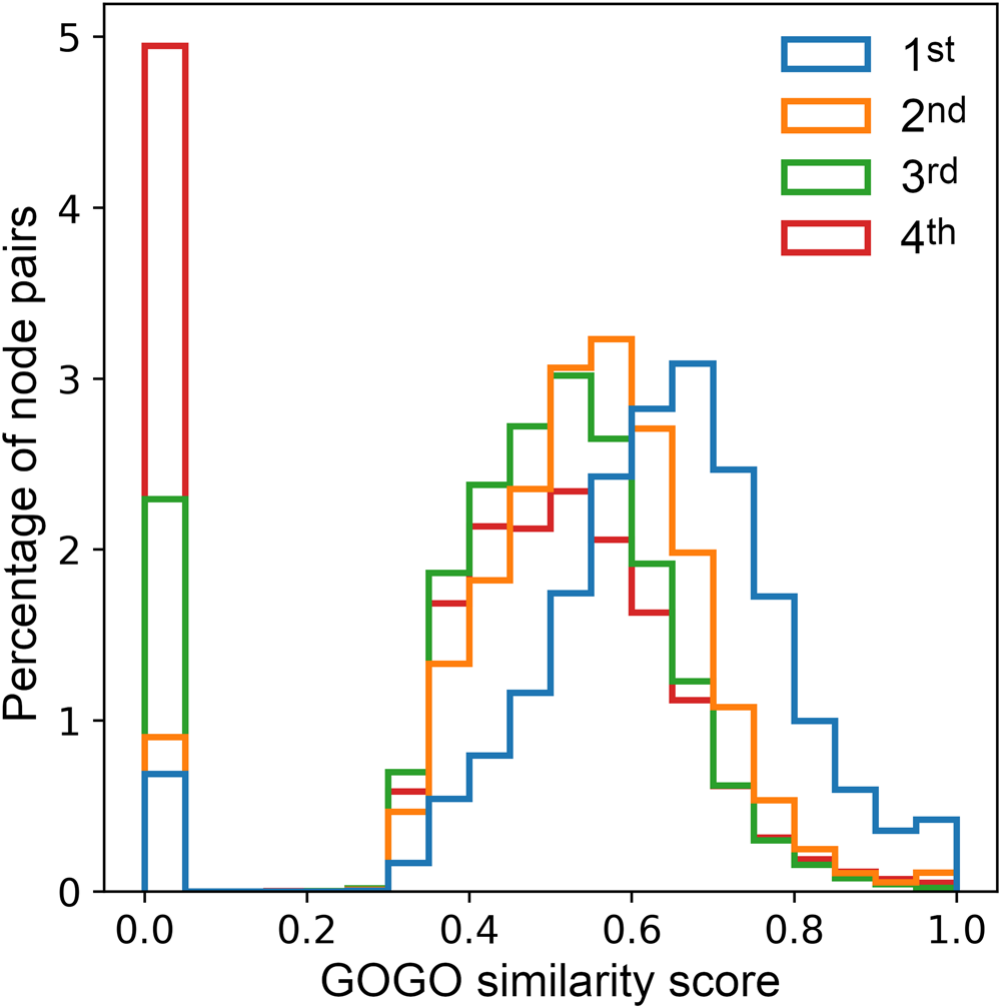
Histogram of the pairwise GOGO similarity scores across the protein-protein interaction network. GOGO similarities are calculated using the biological process ontology for 1st, 2nd, 3rd, and 4th order neighbors in the network.

GOGO is a method to calculate semantic similarities between GO terms using Directed Acyclic Graphs (DAGs)^20^. GO consists of three DAGs created based on molecular function (MF), cellular component (CC), and biological process (BP) ontologies^21^. In order to verify that the network locality is preserved when using GOGO similarities derived from the BP ontology, we first calculated similarity values between 1^st^, 2^nd^, 3^rd^, and 4^th^ order neighbors in the full-size PPI network. Figure 2 shows that GOGO similarities are the highest for the 1^st^ order neighbors and decrease with the increasing order. These results corroborate previous studies demonstrating that the closer the two proteins are in the network the more similar are their biological functions^22^. Next, using GOGO similarities and the hierarchical clustering analysis (HCA), all proteins in the graph were partitioned into 30 (HCA-30), 100 (HCA-100), and 300 (HCA-300) clusters. Only those nodes belonging to the same cluster are allowed to be merged during graph reduction.

**Fig. 3:**
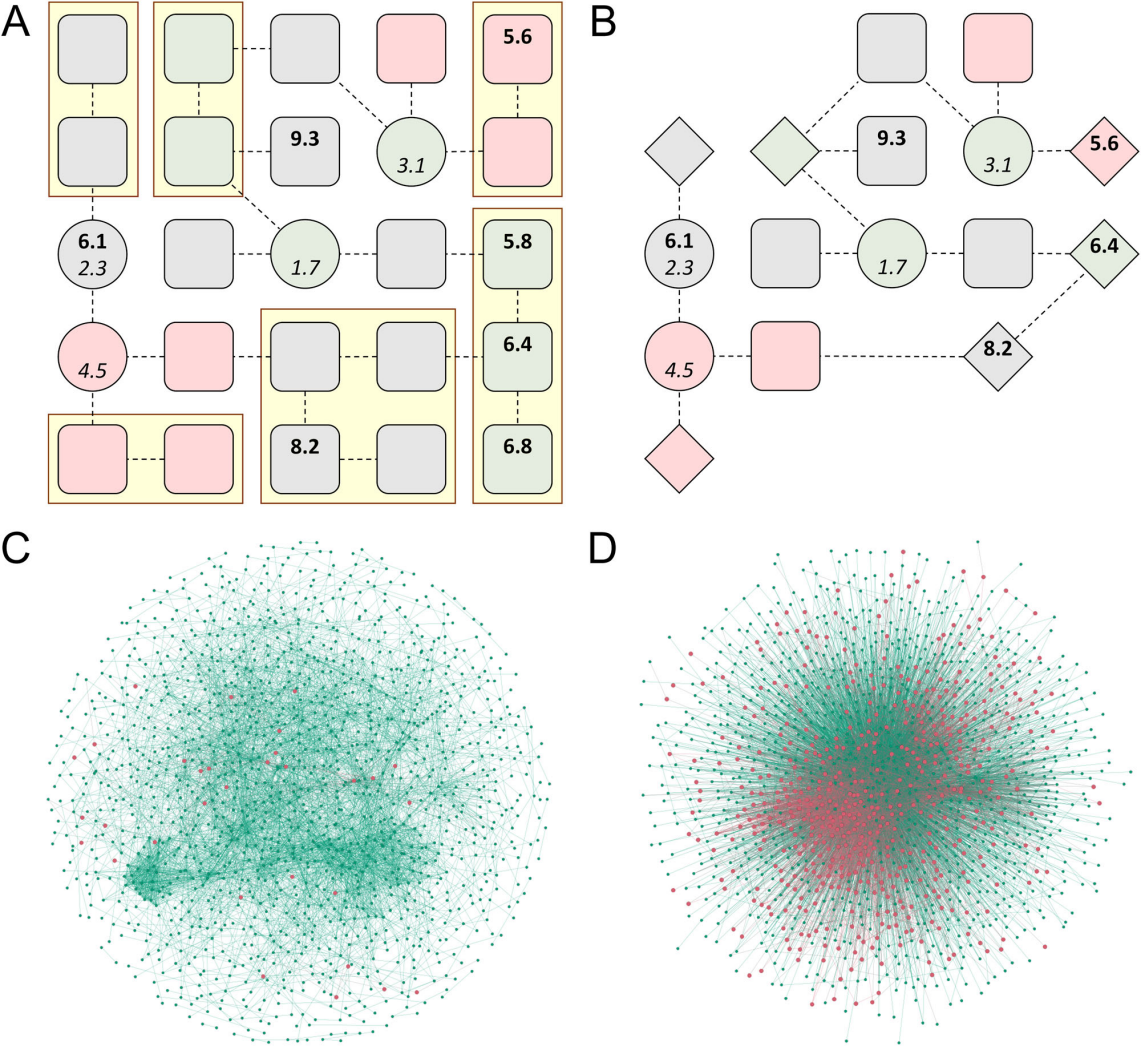
Graph reduction of cancer-specific networks. **A** A schematic of the initial graph with yellow boxes outlining groups of nodes that can be merged by contracting their edges. **B** A schematic graph of the reduced graph in which merged nodes are represented by diamonds. **C** The initial (sub)network for glioblastoma (cell line A172) with red nodes representing kinases and green nodes representing other proteins. **D** The reduced network for glioblastoma colored the same as in (**C**). The network in (**C**) is a randomly sampled subgraph from the original network with the same number of nodes as (**D**).

The reduction procedure for a graph is schematically shown in Fig. 3. An edge may be contracted only if both incident nodes are non-kinases, share the same differential gene expression, and belong to the same GOGO cluster. Yellow squares in Fig. 3A delineate groups of nodes that can be merged by contracting edges connecting them. The resulting reduced graph shown in Fig. 3B has the same number of kinases (circles), but fewer non-kinase proteins (rounded squares and diamonds representing merged nodes). The rationale behind this procedure is not only to reduce the size of a graph, but also to create more diversity among cell lines, which is highly beneficial for further machine-learning applications. Note that the reduction is performed on different cell lines without any drug information because we consider only the differential gene expression, the type of node (kinase or non-kinase protein), and the biological process assignment of nodes, with the latter two properties being independent on the cell line type.

### Analysis of full-size and reduced networks

Compared to the original PPI network (Fig. 3C), the reduced graph (Fig. 3D) has a much higher ratio of kinase (red dots) to non-kinase (green dots) proteins. Table 1 shows that after the reduction, the number of nodes decreased from 19,144 to 1349 and the graph density increased from 0.004 to 0.014, saving computational resources in future graph processing. Further, a higher clustering coefficient for reduced graphs implies that they have better structures in terms of the information exchange than full-size networks. A high maximum betweenness centrality is to be expected since reduced graphs contain a super-hub node connected to many other nodes in the graph. A significant increase of the average betweenness centrality indicates that the information is going to flow more efficiently throughout reduced graphs. Overall, statistics reported in Table 1 demonstrate that in contrast to full-size networks, reduced graphs are compact, yet information-rich offering an effective representation of the biological data for machine-learning applications.

**Table 1:** Properties of full-size and reduced graphs.

Property	Full-size graph	Reduced graphs
Number of nodes	19,144	1349 ± 80
Number of edges	685,198	12,613 ± 608
Average degree	71	19 ± 0.3
Density	0.004	0.014 ± 0.0009
Diameter	8	4.073 ± 0.26
Clustering coefficient	0.287	0.659 ± 0.006
Maximum betweenness centrality	0.021	0.596 ± 0.011
Average betweenness centrality	1.11 × 10^−4^	7.88 × 10^−4^ ± 4.49 × 10^−6^

Statistics are calculated from the graph topology without considering node features. Values for reduced graphs are reported as the average ±standard deviation across the dataset.

**Fig. 4:**
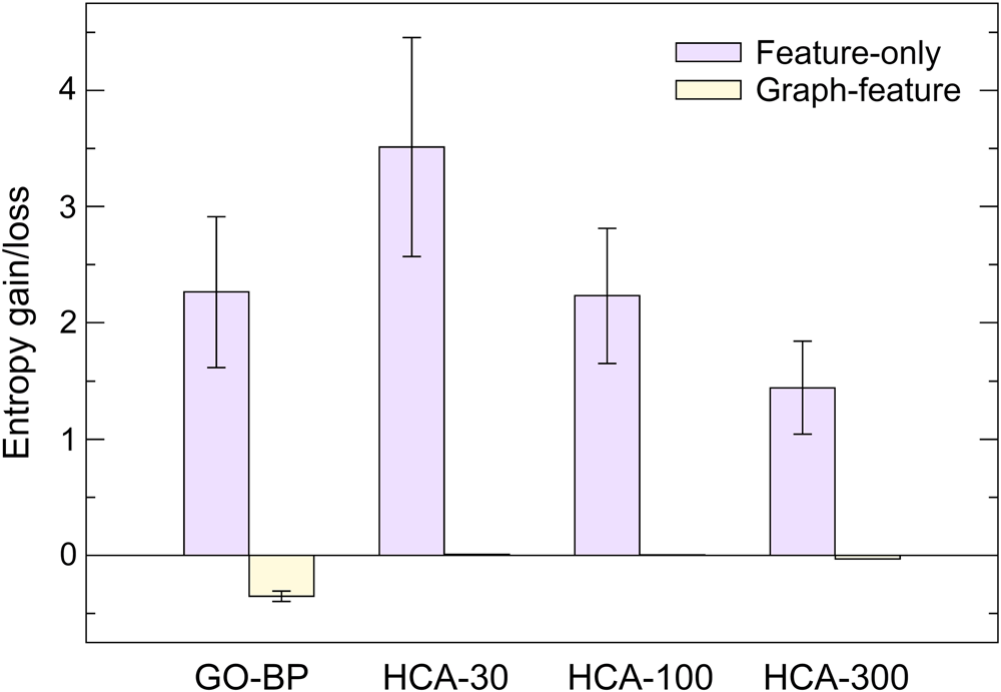
Entropy gain/loss for different reduction schemes. Purple bars represent the Shannon entropy calculated using the feature matrix only, while yellow bars correspond to the graph-feature entropy computed using both feature and topological information of a graph. GO-BP requires that two incident nodes have a common biological process term to be assigned to the same cluster. HCA bars correspond to the clustering using GOGO similarities into 30, 100, and 300 clusters.

The graph reduction procedure greatly increases the diversity of graph topologies and features across all 359 cancer cell lines, while maintaining the important information and biological knowledge in each graph. In order to demonstrate the effectiveness of our reduction scheme, we calculated the information gain/loss after the reduction using the Shannon entropy of features and the graph-feature entropy (see Eq. 3). Ideally, the graph reduction should increase the information content for features without any decrease in the graph-feature information. The information gain/loss is shown in Fig. 4 for several reduction schemes. Although the simplest reduction requiring incident nodes to share at least one GO-BP term increases the feature-only entropy by 2.3 ± 0.6 (green bar), it causes a detrimental decrease of the graph-feature entropy by −0.4 ± 0.04 (red bar). In contrast, HCA increases the feature-only entropy while preserving the valuable graph-feature information. In particular, partitioning nodes into 30 clusters not only yields the highest information gain for features of 3.5 ± 0.9, but also slightly increases the graph-feature entropy. Based on these results and due to the fact that there are actually 30 level-1 biological processes in GO^23^, we incorporated HCA-30 into the graph reduction procedure.

### Comparison of matrix- and graph-based machine-learning approaches

In order to properly evaluate the performance of methods to predict the response of cancer to drugs, we conducted a cross-validation at the tissue level. The entire dataset was first divided into nine groups of different tissues, digestive system, respiratory system, haematopoietic and lymphoid tissue, breast tissue, female reproductive system, skin, nervous system, excretory system, and others. Next, we conducted a 9-fold cross-validation, each time using cancer cell lines from one tissue as a validation set while the remaining cancer cell lines were used for model training. Since cell lines collected from different tissues have different gene expression patterns, this cross-validation scheme eliminates the overlap between training and validation data. In addition, there is also a desired feature variability on account of different gene-disease associations which depend on the cell line and tissue type. Essentially, each fold has entirely different training and validation data.

Table 2 reports the cross-validated performance of matrix- and graph-based algorithms. The first matrix-based approach employs a multilayer perceptron (MLP) to the differential gene expression data along with ligand embeddings. This method represents an appropriate baseline because many available tools reported in the literature utilize similar data frameworks^24^. The cross-validated accuracy of the baseline algorithm is only 0.55 indicating that the model is unable to discover the underlying patterns connecting the genomic data and small molecule descriptors. We also tested another matrix-based approach utilizing the same set of features as that used in the graph-based model. The accuracy of this classifier indeed improved to 0.60 demonstrating that kinase inhibitor profiling and disease-gene associations are more effective compared to ligand embeddings. One possible reason for this improved accuracy is that chemically dissimilar drugs can exhibit similar pharmacological effects in cell lines sensitive to these compounds^25^, which remains undetected by using ligand embeddings.

**Table 2:** Performance of algorithms to predict the response of cancer cell lines to drugs.

Data type	Model	Features	ACC	PPV	TPR	MCC	F-score
Matrix	MLP	DGE, LE	0.55	0.63	0.64	0.27	0.55
Matrix	MLP	DGE, KIP, DGA	0.60	0.60	0.60	0.20	0.60
Matrix	SVM-PCA	DGE, LE	0.61	0.70	0.51	0.10	0.40
Matrix	SVM-PCA	DGE, KIP, DGA	0.62	0.72	0.53	0.16	0.45
Matrix	RF-PCA	DGE, LE	0.42	0.56	0.52	0.06	0.33
Matrix	RF-PCA	DGE, KIP, DGA	0.44	0.56	0.53	0.09	0.39
Graph	WL Tree	DGE, KIP, DGA	0.68	0.67	0.65	0.32	0.65

A graph-based approach is compared to two matrix-based methods. The performance of each algorithm is cross-validated at the tissue level.

*MLP* multilayer perceptron, *SVM-PCA* Support Vector Machines with Principal Component Analysis, *RF-PCA* Random Forest with Principal Component Analysis, *WL Tree* Weisfeiler–Lehman graph kernel, *DGE* differential gene expression, *LE* ligand embeddings, *KIP* kinase inhibitor profiling, *DGA* disease-gene associations, *ACC* accuracy, *PPV* precision, *TPR* recall, *MCC* Matthews correlation coefficient.

In addition to MLP, we tested two other machine-learning approaches, Support Vector Machines (SVM) and Random Forest (RF), both employing the Principal Component Analysis (PCA) to reduce the dimensionality of input feature vectors. Although using SVM-PCA yields a higher accuracy than MLP, the Matthews correlation coefficient^26^ and F-score values are notably lower. These results demonstrate that MLP achieves a robust performance across four confusion matrix categories (true positives, false negatives, true negatives, and false positives), proportionally to the size of positive and negative instances^27^. It is noteworthy that similar to MLP, SVM-PCA and RF-PCA perform better when kinase inhibitor profiling and disease-gene associations are used instead of ligand embeddings. Encouragingly, a graph-based approach employing the WL Tree, a widely adopted graph kernel method for graph machine learning^10^, yields the highest classification accuracy of 0.68, the MCC of 0.32, and the F-score of 0.65, outperforming all matrix-based algorithms. These benchmarking calculations demonstrate that the non-Euclidean representation offers a powerful approach to fully utilize biological data in machine learning.

## Discussion

In this communication, we describe a new procedure to construct cancer-specific biological networks by integrating multiple heterogeneous data, including protein-protein interactions, differential gene expression, gene-disease associations, and kinase inhibitor profiling. In order to improve the effectiveness of machine learning, a graph reduction protocol was implemented to convert initially information-sparse biological networks to information-rich graphs preserving the biological knowledge. In contrast to the original PPI networks that share exactly the same topology, the reduced graphs provide a diverse set of graph topologies across different cell lines. In addition, because of the much smaller size compared to full-size PPI networks, the reduced graphs can be used with any machine-learning model, even those that are computationally expensive. Overall, our procedure to construct cancer-specific molecular networks enables a more efficient learning process on account of the more diverse and information-rich data. This approach improves the performance of machine learning to predict the responsiveness of cancer to pharmacotherapy.

## Materials and methods

### Protein-protein interaction network

Protein-protein interaction data were acquired from the STRING database^28^ that comprises 19,354 human proteins forming 11,355,804 interactions including those identified experimentally and predicted computationally. Each interaction is assigned a confidence score ranging from 150 for low-confidence to 999 for high-confidence interactions. A human PPI network was constructed from confident interactions with a score of ≥500. Single proteins disconnected from the main network and those forming small, isolated networks were removed, resulting in the final PPI network containing 19,144 proteins and 685,198 interactions.

### Differential gene expression data

Original gene expression data are available from the Cancer Cell Line Encyclopedia (CCLE) project comprising a detailed genetic characterization of a large number of cancer cell lines^29^. We obtained the curated CCLE data from Harmonizome, a comprehensive repository of processed genomics, proteomics, epigenomics, transcriptomics, and metabolomics data^30^. The differential gene expression (DGE) data contain 18,022 genes, 1035 cancer cell lines, and 749,551 gene-cell associations categorized as down-, up-, and normally regulated with respect to the expression level in healthy cells.

### Kinase inhibitor profiling

Inhibitor profiling refers to a large-scale experimental measurement of the activity of an inhibitor against a panel of target proteins. The kinase inhibitor profiling (KIP) data used in this study were collected and curated by Team-SKI^31^. The activity is reported as a pIC_50_ value, which is the negative logarithm of the half-maximal inhibitory concentration (IC_50_) measuring the potency of a compound in inhibiting a specific biochemical function. The cutoff for pIC_50_ values was set at 6.3 corresponding to the IC_50_ of 500 nM. The Team-SKI dataset contains 49,348 small molecules tested against 411 protein kinases.

### Disease-gene associations

Disease-gene association (DGA) scores were obtained from two sources, DISEASES^32^ and DisGeNET^33^. The DISEASES database integrates evidence on associations collected through automatic text mining, manually curated from biomedical literature, cancer mutation data, and genome-wide association studies. It contains 8330 diseases and 20,715 genes with association scores ranging from 1 to 10. The DisGeNET database provides information on associations pulled from various repositories including Mendelian, complex and environmental diseases, and integrated using gene/disease vocabulary mapping and the DisGeNET ontology. It comprises 24,166 diseases and 17,545 genes with association scores ranging from 0.01 to 1.

### Data integration

Data integration was performed by mapping DGE, KIP, and DGA values directly onto the PPI network. Gene expression data contains normalized scores for 18,022 genes, whereas the PPI network comprises 19,144 proteins. The missing values for nodes in the graph were replaced by median scores calculated over 1st order neighbors. The kinase component of the PPI network was determined by running Basic Local Alignment Search Tool (BLAST)^34^ against known human kinases^35^. Using a similarity threshold of 95% identified 508 kinases. pIC_50_ values are available from the Team-SKI database for 411 kinase proteins in our network and 29 small molecules that are also present in the growth rate inhibition data from LINCS. The Disease Ontology ID (DOID) and the Concept ID for each cancer cell line were identified with the Cellosaurus resource portal^36^, and mapped to, respectively, DISEASES and DisGeNET databases in order to assign disease-gene association scores to nodes in the PPI network. After integrating all data and removing cases that could not be mapped, the final dataset contains annotated graphs for 3,549 combinations of 359 cell lines and 29 drugs.

### Growth rate inhibition data

Proliferation is measured by GR_50_ and GR_max_ indices quantifying the value of growth rate inhibition (GR) based on time course and endpoint assays^37^. GR_50_ is the concentration of a drug at which GR is 0.5, whereas GR_max_ is the maximum measured GR value. In this study, we employ GR_max_ values for the following dose-response datasets: Broad-HMS LINCS Joint Project, LINCS MCF10A Common Project, HMS LINCS Seeding Density Project, MEP-HMS LINCS Joint Project, Genentech Cell Line Screening Initiative, and Cancer Therapeutics Response Portal^37^. The benchmarking dataset comprises 2124 positive instances of cell line-drug combinations with negative GR_max_ values signifying a cytotoxic response, and 1425 negative instances with positive GR_max_ values signifying a cytostatic response.

### Graph partitioning

The latest release of the GO database contains 29,698 BP, 11,147 MF, and 4201 CC ontology terms^21^. Each DAG has its own hierarchy maintained by the domain-centric Gene Ontology (dcGO). According to the dcGO database, the numbers of level-1 nodes are 30, 15, and 22 for BP, MF, and CC, respectively^23^. Since our goal was to assign nodes to biological pathways, all-against-all semantic similarities between GO terms of PPI network nodes were calculated with the GOGO software based on the BP-DAG topology. The matrix of GOGO scores was then subjected to a hierarchical clustering analysis, which builds nested clusters by successively merging and splitting clusters^38^. There are two types of hierarchical clustering, agglomerative (bottom-up) and divisive (top-down). Agglomerative methods merge observations as moving up the hierarchy while divisive methods split the observations as moving down the hierarchy. Here, we employed the agglomerative HCA rather than other commonly used clustering methods, such as k-means^39^, DBSCAN^40^, and affinity propagation^41^ because this technique is the most appropriate for GOGO semantic similarities and we found empirically that the clustering results represent well different levels of biological process in GO.

### Graph statistics

*Average degree* of a graph is calculated by averaging the degrees of all nodes, defined as the mean number of connections to other nodes in the graph.

*Density*, $\rho _G$, is defined as the ratio of the number of edges and the number of possible edges. For an undirected graph, it is calculated as

1 $$\rho _G = \frac{{2\left| E \right|}}{{\left| V \right|\left( {\left| V \right| - 1} \right)}}$$

where $|E|$ is the number of edges and $|V|$ is the number of nodes. Since the information is propagated more efficiently across dense graphs, increasing the graph density is generally beneficial for graph learning algorithms, such as GNN.

*Diameter* is the longest shortest path between any two nodes in a graph^19^, i.e. the longest distance one must traverse from one node to reach another node. This is an important property because it directly informs the design of a graph model.

*Clustering coefficient* measures the tendency of nodes to form clusters. Most real-world graphs, such as social, powerline, citation, language, food, economic, and biological networks, tend to form communities, which are characterized by a high density of ties^42^. The higher the clustering coefficient, the more likely nodes are to form communities resulting in a shift of the information exchange from global to local.

*Betweenness centrality* of a node is the sum of the fraction of all-pair shortest paths passing the node^43^. Essentially, this property evaluates the amount of information flowing through nodes, i.e., a high betweenness centrality signifies more information flowing through a graph. Further, those nodes with a high information flow interact more frequently with other nodes, maintaining a significant control over the entire network.

### Graph-feature entropy

A graph reduction basically compresses the information in a graph. However, as all lossy compression schemes, some information will be lost during the reduction. In order to measure how much meaningful information is lost or gained as opposed to the loss/gain of the redundant or irrelevant information, we introduce a concept of the graph-feature entropy. Briefly, the graph-feature entropy $S$ of a graph $G$, $S_G$, is based on the Shannon entropy^40^ defined as:

2 $$S_{{G\,\,}^{\underline{\underline {{{{\mathrm{def}}}}}}}}- \mathop {\sum}\limits_j p \left( {z_j} \right)\log p\left( {z_j} \right)$$

where $z_j$ is the column vector of the feature matrix filtered by the graph Laplacian, which corresponds to the *j*-th feature of all nodes in the graph. Using features filtered with the graph Laplacian effectively combines both feature and topological information of a graph providing a useful measure of the information content in featured graphs.

Next, we calculate the information gain/loss after the reduction, δ, using the Shannon entropy of features and the graph-feature-entropy:

3 $$\delta = \frac{{S_{{{{\mathrm{reduced}}}}} - S_{{{{\mathrm{original}}}}}}}{{S_{{{{\mathrm{original}}}}}}}$$

where $S_{{\rm{original}}}$ is the entropy (either feature-only or graph-feature) of the original graph and $S_{{\rm{reduced}}}$ is the corresponding entropy of the reduced graph.

### Matrix-based methods to predict drug response

The rows of input matrices are gene products, and the columns contain various features. The baseline matrix includes only the DGE data, and after flattening to a 19,144-element vector, it is concatenated to a 300-element vector of ligand embeddings (LE) computed by Mol2vec^41^ for the input drug. The baseline classifier utilizing 19,444-element input feature vectors represents an archetypal approach to predict a drug response based solely on the gene expression changes and the drug chemical structure. The second method utilizes a full input matrix of 19,144 gene products × 3 features, DGE, KIP, and DGA, flattened to a 57,432-element vector. This approach is a matrix-based equivalent of the graph-based method utilizing exactly the same input features. Both matrix-based classifiers employ an MLP with an input layer of either 19,444 units for the baseline or 57,432 units for the graph-equivalent method, three hidden layers comprising 1024, 512, and 256 units, and a 2-unit output layer returning probabilities for the input drug to be effective and ineffective against the cell line.

### Graph-based method to predict drug response

The graph-based approach devised in this study employs the WL graph kernel, also known as WL Tree classifier^10^, to the reduced representations of cancer-specific PPI networks comprising DGE, KIP, and DGA data. WL Tree is a widely adopted method for learning from graphs with discrete node labels. A key component of this algorithm is a rapid feature extraction employing the WL test of isomorphism on graphs. Briefly, WL Tree iteratively maps the original node labels to new node labels based on the topological structure of the graph. The new node labels are then compressed and integrated with the original node labels in order to form a vector representation for the graph. The implementation of WL Tree used in this study was previously demonstrated to outperform other graph kernels on several graph classification benchmark datasets in terms of accuracy and runtime.

## Data Availability

Data generated in this study are freely available to the academic community at https://osf.io/dzx7b/.
